# 6DOF Aircraft Landing Gear System with Magnetorheological Damper in Various Taxing and Touchdown Scenarios

**DOI:** 10.3390/mi16030355

**Published:** 2025-03-20

**Authors:** Quoc-Viet Luong, Quang-Ngoc Le, Jai-Hyuk Hwang, Thi-My-Nu Ho

**Affiliations:** 1 Faculty of Mechanical Technology, Ho Chi Minh City University of Industry and Trade, 140 Le Trong Tan Street, Tay Thanh Ward, Tan Phu District, Ho Chi Minh City 700000, Vietnam; vietlq@huit.edu.vn; 2 Industrial Maintenance Training Center, Ho Chi Minh City University of Technology, 268 Ly Thuong Kiet Street, District 10, Ho Chi Minh City 700000, Vietnam; lqngoc@hcmut.edu.vn; 3 Vietnam National University Ho Chi Minh City (VNU-HCM), Linh Trung Ward, Thu Duc City, Ho Chi Minh City 700000, Vietnam; 4 School of Aerospace and Mechanical Engineering, Korea Aerospace University, Goyang-si 10540, Republic of Korea; jhhwang@kau.ac.kr

**Keywords:** aircraft landing gear, aircraft taxing, aircraft landing, neural network control, skyhook control, magnetorheological damper

## Abstract

This manuscript presents a new approach to describe aircraft landing gear systems equipped with magnetorheological (MR) dampers, integrating a reinforcement learning-based neural network control strategy. The main target of the proposed system is to improve the shock absorber efficiency in the touchdown phase, in addition to reducing the vibration due to rough ground in the taxing phase. The dynamic models of the aircraft landing system in the taxing phase with standard landing ground roughness, one-point touchdown, two-point touchdown, and third-point touchdown are built as the first step. After that, Q-learning-based reinforcement learning is developed. In order to verify the effectiveness of the controller, the co-simulations based on RECURDYN V8R4-MATLAB R2019b of the proposed system and the classical skyhook controller are executed. Based on the simulation results, the proposed controller provides better performance compared to the skyhook controller. The proposed controller provided a maximum improvement of 16% in the touchdown phase and 10% in the taxing phase compared to the skyhook controller.

## 1. Introduction

The damper system in a road vehicle reduces the vibration during the rough ground to improve the passenger’s comfort. Different from the damper system in the car, the aircraft’s landing gear system not only improves the passenger’s comfort during the taxing phase, but also absorbs the landing energy during landing [1]. Moreover, the landing gear system must improve safety during hard landing with various landing scenarios. So, the landing gear system must be able to adjust its characteristics in various landing conditions. The conventional aircraft landing gear system is equipped with passive dampers that have transformed their behaviors by a small orifice [2,3]. This landing system only provides a good performance in specified landing conditions; therefore, many researchers have been attempting to develop new aircraft landing gear systems.

An active landing gear system [4] and a semi-active landing gear system [5] have been invented to replace the conventional landing gear system. The active landing gear system has a huge range of controllable damping forces; however, it requires a strong and reliable hydraulic system that increases the cost of equipment [6]. Moreover, if the electrical part does not work well, the system will fail. On the other hand, the semi-active damper has both the advantages of the active damper and the passive damper [7]. When the electrical component breaks, the semi-active damper switches to the passive damper, and the semi-active damper can modify its characteristics by changing the voltage or electrical current [8,9].

The magnetorheological (MR) damper, which is a semi-active damper, is highly controllable because it can change to be semi-solid from a fluid state with an increase in the applied magnetic field [10]. Many researchers have attempted to integrate the negative stiffness mechanism into aircraft landing gear systems by using an MR damper. For example, Byung-Hyuk Kang et al. [11,12,13] designed a new magnetorheological aircraft main landing gear system and comprehensively tested with mathematical modeling and drop testing for the evaluation of the landing efficiency and a controller based on the skyhook controller was developed to improve the landing performance. Jo et al. [14] developed a prototype MR damper landing gear, a two-degree-of-freedom mathematical mode. The authors also verified the accuracy of the model by comparing it with the drop test experiment. Dong et al. [15] built a mathematical model of the aircraft landing gear with an MR damper and developed the linear active disturbance rejection control technique to prevent the shimmy phenomenon on a nose landing gear. Hao et al. [16] adopted this to optimize the structural parameters of an MR landing gear system for a UAV cluster combat.

In all the previous research, the researchers only focused on the behavior of the landing gear either in the touchdown phase [17,18] or the taxing phase [19], as can be seen in Table 1. In the touchdown phase, the main goal of the landing gear system is to reduce the landing force attached to the aircraft body frame as much as possible, and the shock absorber efficiency is the factor used to verify the effectiveness of the system [20]. The single landing gear drop test experiment [21] or simulation [22] was built and executed to determine the shock absorber efficiency. In the taxing phase, the main function of the aircraft landing gear system is to reduce the aircraft body’s acceleration as much as possible for the passenger’s comfort; the root mean square of the acceleration is the key factor in developing the controller in the taxing phase [23]. Developing a prototype experiment that performs both the touchdown phase and taxing phase is expensive and time-consuming, and building a model that simulates both phases of the landing process is challenging; thus, few studies have adopted the MR landing gear system considering both the taxing phase and the landing phase.

The main contribution of this manuscript is to adopt a landing gear system equipped with an MR damper that can improve the shock absorber efficiency in the touchdown phase and reduce aircraft acceleration during taxing mode. In this study, the mathematical model is presented and analyzed, and a multi-body dynamics model is built using RECURDYN V8R4 software. A new landing gear performance, which is a combination of the shocked absorbed efficiency and the root mean square, is given to verify the landing gear system in the whole landing gear process. An intelligent control based on reinforced learning is developed. The structure of the paper is as follows: Section 2 shows the detail of the structure of the aircraft landing gear system equipped with MR damper; Section 3 adopts the mathematical model of the touchdown process; Section 4 details the taxing phase in RECURDYN software; Section 5 presents the structure of the proposed controller and the Q-learning algorithm; Section 6 shows the simulations and discussion; and the conclusion is outlined in Section 7.

## 2. Aircraft Landing Gear System

**Figure 1:**
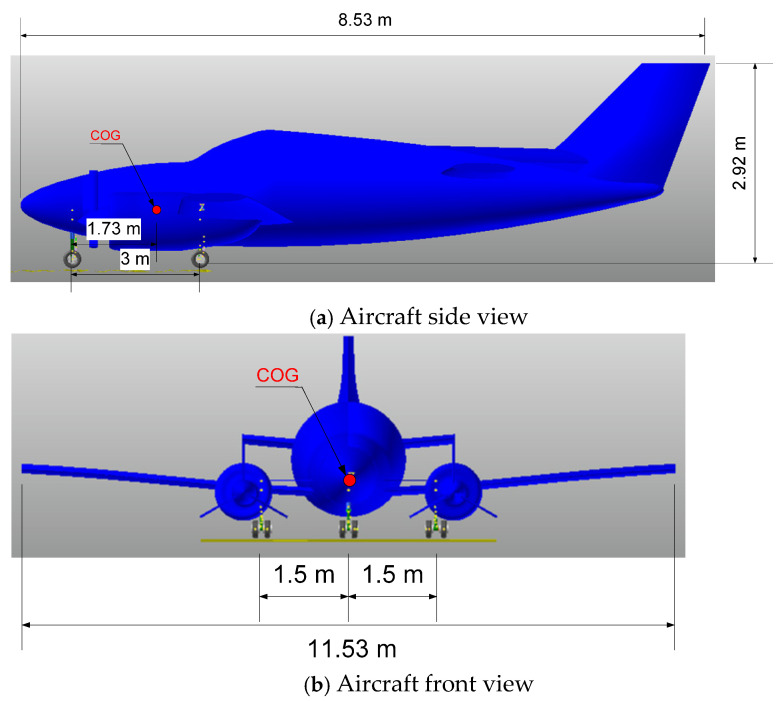
Front view and side view of aircraft.

**Figure 2:**
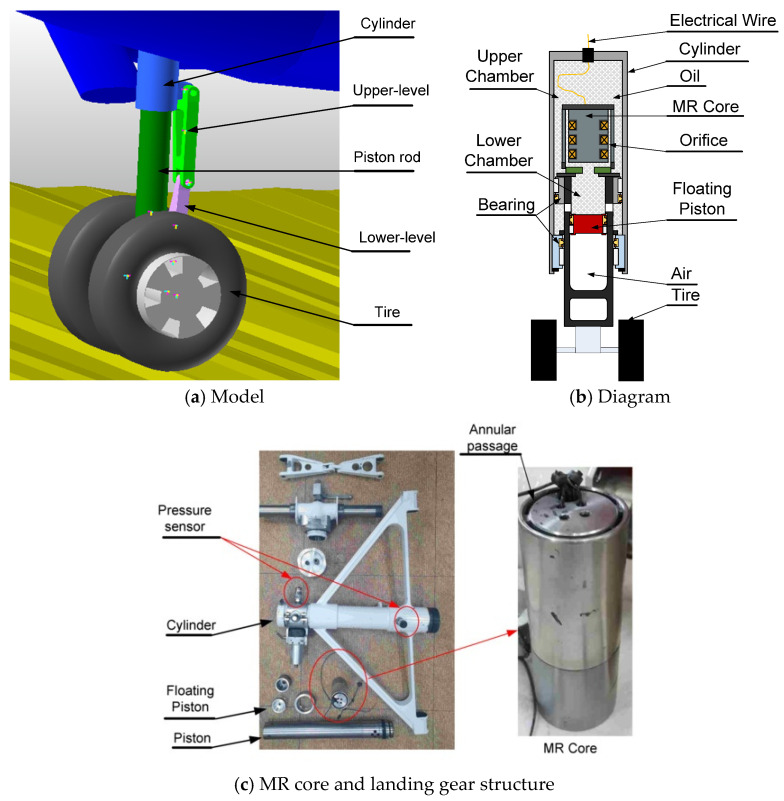
Single landing gear system.

The aircraft referenced in this research is a light twin-engine piston aircraft called the Beechcraft Baron [30]. This aircraft was drawn based on its dimensions with a scale of 1:1 by using RECURDYN software, as can be seen in Figure 1. In this model, the 3-DOF aircraft body was assumed to be rigid and it was set up with initial parameters such as *M*, *v*_0_, *Ixx*, and *Iyy*, as shown in Table 2. In our previous research, we developed the drop test experiment of a single landing gear system equipped with an MR damper [14,21,28]. In this system, a single landing gear is composed of two wheels, a cylinder, a piston, and a pair of levels, as detailed in Figure 2a. A pair of levels maintain a strange direction to avoid the shimmy phenomenon. A cylinder is fixed into the aircraft body, while the piston can translate smoothly into the cylinder which has 1-DOF. So, the proposed system involves 6-DOF.

In the previous research, the single landing equipped with an MR damper was designed and verified by drop-test experiments, as can be seen in [13,14,21]. In this study, the design of the single landing gear continuously is used. The weight of each landing gear is around 20 kg. The cross-section of the landing gear is simplified in Figure 2b. The primary part of the landing gear consists of a gas accumulator, a cylinder, MR fluid, a floating piston, and a piston. The MR fluid, MRF-140CG [31], which is fully injected into the MR damper, is separated into upper and lower hydraulic chambers by the piston head. This fluid is forced from one chamber through the holes to the opposite chamber by the movement of the piston when the landing gear touches the ground. Frictional forces are generated within the vital fluid, as well as between the fluid and the piston. Therefore, when the landing gear lands, the landing energy can be easily absorbed. To compensate for the volume of the piston head due to the piston movement, compressed air is collected inside the piston. In addition, it acts as a spring, retaining the excess energy during the compression phase and releasing the energy during the subsequent extension phase. The magnetic circuit is located in the MR core, which is located inside the piston head. Three electromagnets are used in this design to generate strong magnetic fields. An electrical cable that exits the damper through a hole at the top of the cylinder carries electrical current from the external electric board to the MR core. To lessen the friction force between the piston and the bearings, the landing gear features two wheels that are equally positioned.

**Figure 3:**
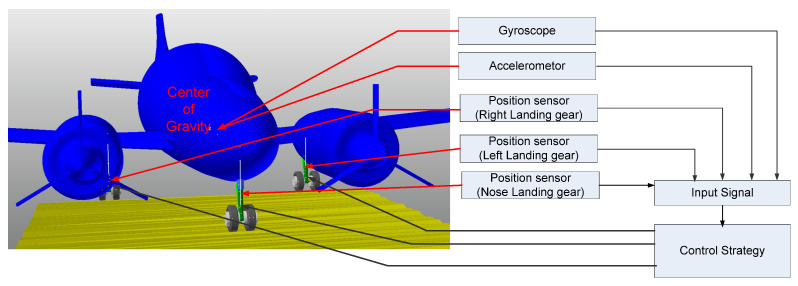
Aircraft landing gear control system.

Our aircraft landing gear system’s control architecture is depicted in Figure 3. It is believed that each landing gear has simply a position sensor that measures relative motion (i.e., the stroke of the cylinder and piston) due to weight restrictions and measuring instrument costs. In addition to providing the aircraft’s acceleration and three-axis rotation rates at the center of gravity, the aircraft control system also has gyroscopes and accelerometers that can be utilized to regulate the landing gears. After obtaining the sensor signals, our system’s control strategy calculates the necessary electrical current for each landing gear. The damping force needed to claim the control objective is then produced by applying the electrical current to the MR dampers. Thus, the damping force (*F_d_*) of the landing gear involves viscous force (*F_v_*), pneumatic force (*F_a_*), and MR force (*F_MR_*), as presented below [21]:

(1) $$F_{d}=F_{v}+F_{a}+F_{MR}$$

(2) $$F_{v}=C\dot{s}$$

(3) $$F_{a}=A_{p}p_{0}{\left(\frac{V_{0}}{V_{0}-A_{p}s}\right)}^{n}$$

(4) $$F_{MR}=f\left(u,\dot{s}\right)$$

**Figure 4:**
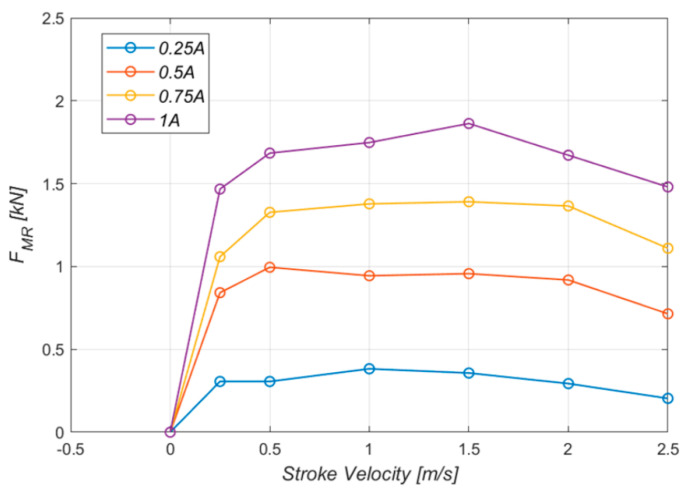
Controlled force FMR.

In the previous research [21], the control force was found in the drop test experiment by varying the current applied to the MR damper core from 0 A to 1 A at 0.25 A intervals. The results are shown in Figure 4. The control damping force increased as the current increased; at a current of 1 A, the MR force reached a maximum value of 1.7 kN.

## 3. Touchdown Phase

**Figure 5:**
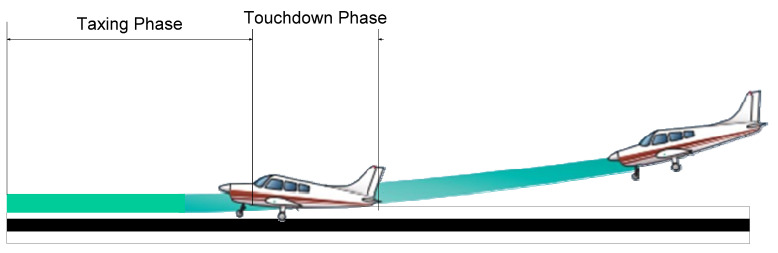
Aircraft landing phase.

**Figure 6:**
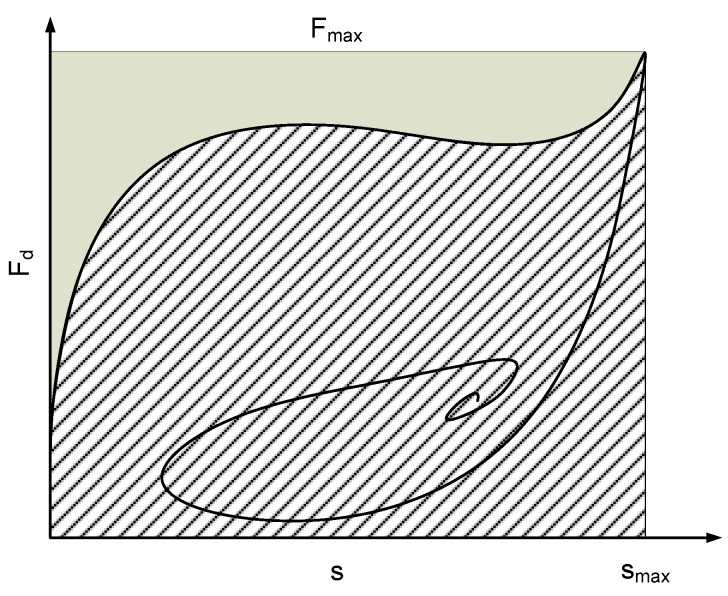
Shock absorber efficiency.

The airplane gently resting on the landing surface is known as a touchdown, as detailed in Figure 5. In order for the aircraft to land on the main gear at roughly stalling speed, the roundout and touchdown should be performed with the engine running and the aircraft at the lowest controllable velocity. Any required back-elevator pressure is applied when the aircraft settles to achieve the ideal landing attitude [32]. The aircraft makes its first contact with the ground in a very short time. Following [20], the touchdown process is determined by the time that the aircraft makes the first stroke cycle. In order to verify the performance of the landing gear, the shock absorber efficiency is defined as:

(5) $$\eta =\frac{\int_{0}^{s_{max}\;}F_{d}ds}{\max\left(F_{d}\right)\max\left(s\right)}$$

where *s*_max_, *F*_max_ are the maximum stroke and damping force, respectively, as can be seen in Figure 6. Based on FAR part 25 [33], the minimum value of shock absorber efficiency of the landing gear in the landing gear shock absorption tests is 0.7. If the shock absorber efficiency is higher, it performs better.

Defend on the number of landing gears that make contact with the ground that defined three main landing cases: three-point touchdown, two-point touchdown, and one-point touchdown.

### 3.1. Three-Point Touchdown

**Figure 7:**
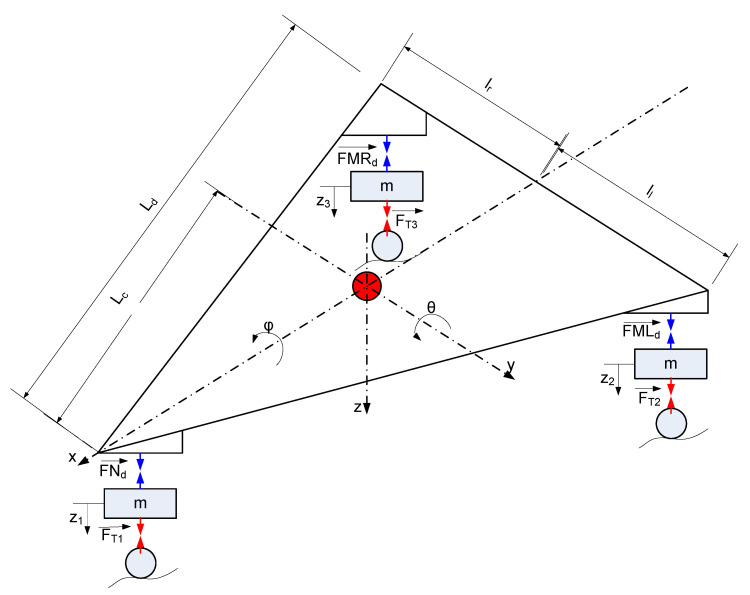
Three-point landing diagram.

In the three-point touchdown case, the aircraft makes contact with the ground by using both three main landing gears. In general aircraft, it is very difficult to make both three landing gears touch the ground at the same time due to rough ground, pilot control, environment. The free-body diagram of the aircraft landing gear system is shown in Figure 7, and the main body has three restricted directions, which are z, pitch (φ), and roll (θ). And their pistons are translated in the z-direction of the cylinder. In this research, yaw motion and break force are assumed to be neglected. Thus, the whole aircraft landing gear system involves 6-DOF. The motion equation of the aircraft landing gear system is given in Equations (6)–(11).

(6) $$I_{xx}\ddot{\varphi }+FML_{d}\;l_{l}-FML_{d}\;l_{r}=0$$

(7) $$I_{yy}\ddot{\theta }-FN_{d}\;l_{a}+\left(FML_{d}+FMR_{d}\right)\;l_{b}=0$$

(8) $$M\ddot{z}=W_{z}-FML_{d}-FMR_{d}-FN_{d}$$

(9) $$m{\ddot{z}}_{1}+FN_{d}-F_{T1}=mg$$

(10) $$m{\ddot{z}}_{2}+F{ML}_{d}-F_{T2}=mg$$

(11) $$m{\ddot{z}}_{3}+F{MR}_{d}-F_{T3}=mg$$

where *W_z_* is the aircraft gravity force; *FN_d_*, *FML_d_*, and *FMR_d_*, are the damping force following Equation (1) of nose landing gear, left landing gear, and right landing gear, respectively; *F_T_*_1_, *F_T_*_2_, and *F_T_*_3_ are presented as the reaction tired force of nose landing gear, left landing gear and right landing gear, respectively, as can be given by:

(12) $$F_{T1}=k_{T}z_{1}^{\prime };\;F_{T2}=k_{T}z_{2}^{\prime };\;F_{T3}=k_{T}z_{3}^{\prime };$$

where $z_{1}^{\prime };\;z_{2}^{\prime };\;and\;z_{3}^{\prime }$ are presented as the tire’s reflection of the nose landing gear, left landing gear, and right landing gear, respectively.

### 3.2. Two-Point Touchdown

**Figure 8:**
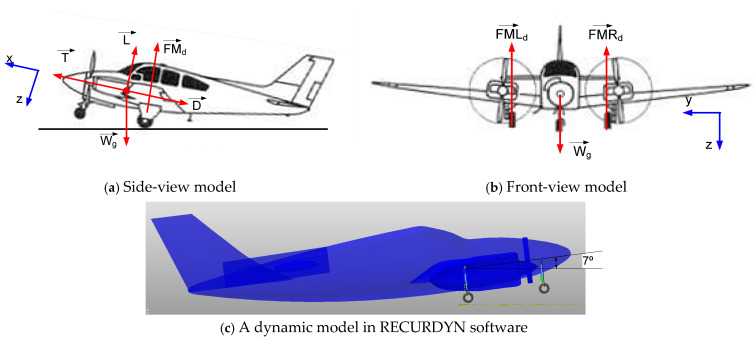
Two-point landing gear model.

In general, an aircraft makes contact with the ground by two main landing gears (left and right main landing gear). The aircraft pitch angle gradually decreases by maintaining the acting engine. After the landing energy is fully absorbed, the nose landing gear contacts slowly with the ground. In a hard landing, the aircraft drops freely without helping the engine; so, the engine thrust (T), lift force (L), and draft force (D) are assembled to be neglected, as can be seen in Figure 8. Thus, Equations (6)–(8) become:

(13) $$I_{xx}\ddot{\varphi }+FML_{d}\;l_{l}-FML_{d}\;l_{r}=0$$

(14) $$I_{yy}\ddot{\theta }-FN_{d}\;l_{a}+\left(FML_{d}+FMR_{d}\right)\;l_{b}=0$$

(15) $$M\ddot{z}-FN_{d}-FML_{d}-FMR_{d\;}=0$$

### 3.3. One-Point Touchdown

**Figure 9:**
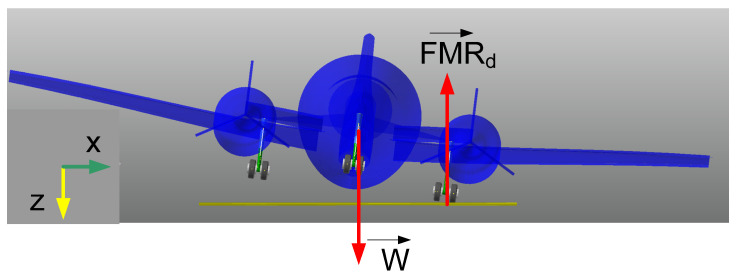
One-point landing gear.

During hazardous conditions, the aircraft only makes contact with the ground with one main landing gear, as can be shown in Figure 9. If the aircraft lands by nose landing, the aircraft is unstable and destroyed. In this study, the aircraft is supposed to make contact with the ground by using the main right landing gear. So, Equations (6)–(8) can be given as:

(16) $$I_{xx}\ddot{\varphi }-FMR_{d}\;l_{r}=0$$

(17) $$I_{yy}\ddot{\theta }+\left(FMR_{d}\right)\;l_{b}=0$$

(18) $$M\ddot{z}-FMR_{d}=0$$

## 4. Taxing Phase

**Figure 10:**
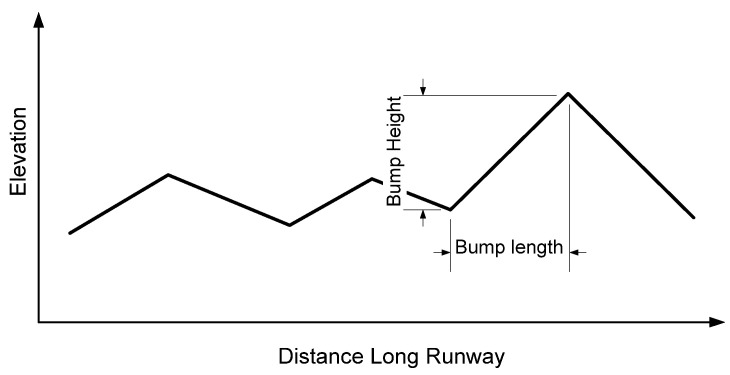
Definition of ground roughness.

**Figure 11:**
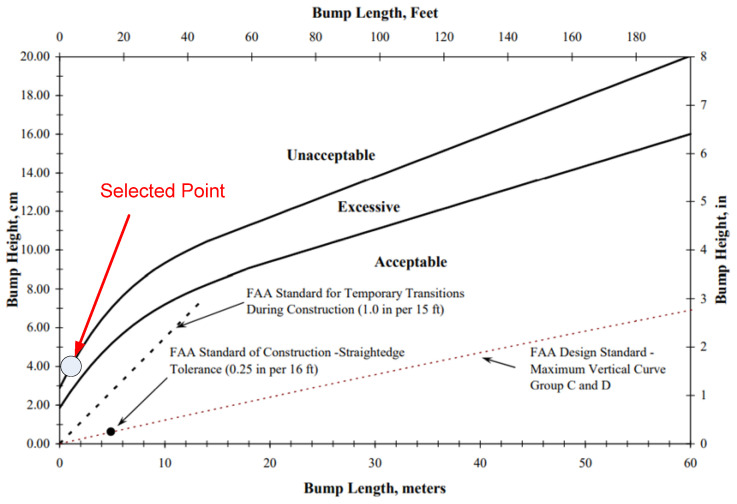
Standard of the ground roughness.

**Figure 12:**
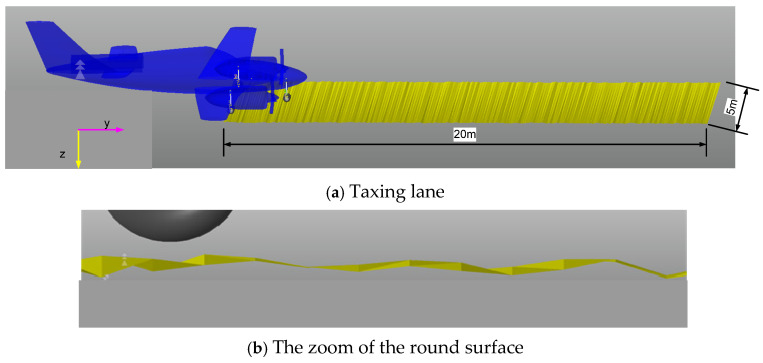
Taxing surface of the ground lane.

During the taxing phase, the aircraft contacts the ground with landing gears, and there are no sink speeds. In order to stabilize the aircraft, the rough ground is not too big. The roughness of the ground plane is defined by two factors which are bump length and bump height, as detailed in Figure 10. Following the FAA standard [34], the values of bump length and bump height are shown in Figure 11. In this research, the maximum value of bump length is 4 cm, corresponding with the bump length being 100 cm. In RECURDYN software, the lane surface is created by associating tiny rectangles with random points that satisfy the maximum bump length, as can be seen in Figure 12. The length and the width of the lane are 20 m and 5 m, respectively.

In the taxing phase, the main target of the landing gear system is to reduce the vibration of the aircraft to improve customer comfort. So, the root mean square (RMS) of the aircraft body is the most popular factor used to verify the vibration of the system, as detailed below:

(19) $$RMS\left(\ddot{z}\right)=\frac{1}{n_{step}}\sqrt{\sum_{i=1}^{n_{step}}\left({\left(\ddot{z}\left(i\right)\right)}^{2}\right)}$$

where *n_step_* is the number of sample signals; $\ddot{z}$ is the aircraft body’s acceleration. The system performs better if it produces a smaller value of RMS.

## 5. Q-Learning Neural Network Controller

**Figure 13:**
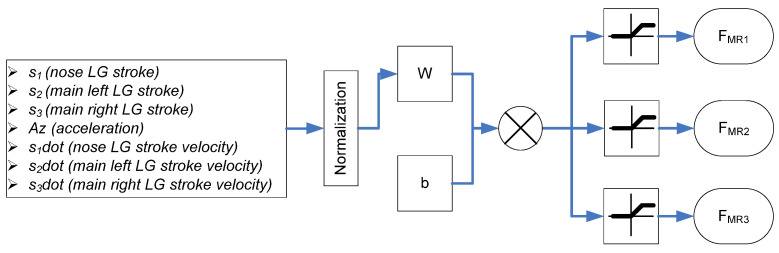
Neural network structure.

In the previous research [21], a supervised learning neural network for a single MR landing gear system was designed based on drop test experiment data. Although the neural network only needs one layer with a weight matrix (*W*) and a bias vector (*b*); it works very well for a single landing gear during touchdown. In this research, we developed the proposed neural network structure based on the neural network structure in the previous research involving more input signals and more output signals. The structure of the neural network is shown in Figure 13. There are eight input signals, which are the stroke signal from each landing gear’s potential sensor (*s*_1_, *s*_2_, *s*_3_); derivate of these signals (*s*_1_*dot*, *s*_2_*dot*, *s*_3_*dot*); and the aircraft body’s accelerometer (*Az*). There are three output signals, which are three electrical currents applying to the nose landing gear (*F_MR_*_1_), the left main landing gear (*F_MR_*_2_), and the right main landing gear (*F_MR_*_3_).

In order to find the optimal value of *W* and *b* or training the neural network, the reinforcement learning method based on the Q-learning algorithm is applied, as shown below [35]:

Initialization:

(20) $$\begin{array}{c} W_{f}=0;\;b_{f}=0;\;Q(W_{f},\;b_{f})=0;\\ G(W_{f},\;b_{f})=G_{\mathrm{Passive}}\\ N=0\\ P=0.5\\ N_{\mathrm{repeat}}=0 \end{array}$$

While looped until *N_repeat_* = 20

(21) $$W,\;b=\left\{\begin{array}{c} W_{f},\;b_{f}\;with\;probability\;1-P\\ (W_{f},\;b_{f})+Q(W_{f},\;b_{f})\times rand\left(\left[0\;1\right]\right)\;with\;probability\;P \end{array}\right.$$

$$\begin{array}{l} \mathrm{If}\;G\left(W,b\right)<G\left(W_{f},b_{f}\right):\\ \;W_{f},\;b_{f}=W,b\\ \;R=0\\ \;\mathrm{else}:\\ \;R=100\\ \;N\mathrm{repeat}=N\mathrm{repeat}+1 \end{array}$$

(22) $$\begin{array}{l} \;N=N+1\\ Q(W_{f},\;b_{f})\;=\;Q(W_{f},\;b_{f})\;+\;\left(R-Q(W_{f},\;b_{f})\right)/N \end{array}$$

In this algorithm, (*W_f_, b_f_*) is the value of the weight matrix and bias vector at the final stage. And the *G*(*W_f_*, *b_f_*) represents the goal’s value of the landing gear system corresponding to the value of *W_f_* and *b_f_*, which means the performance of the landing gear system in certain scenarios. As mentioned in the previous chapter, there are two different performance factors used to verify the efficacy of the landing gear system which are shock absorber efficiency and RMS. From Equation (5), the reduction in max(*F_d_*) could raise the η. From Equation (8), the max(*F_d_*) could be calculated as:

(23) $$\max\left(F_{d}\right)=W_{g}-M\min\left(\ddot{z}\right)$$

So, instead of max(*F_d_*), the $\min\left(\ddot{z}\right)$ is used to check the landing gear performance. Thus, the goal of the landing gear system can be given below:

(24) $$Minimum=>G=\min\left(\ddot{z}\right)\;+RMS(\ddot{z})$$

**Figure 14:**
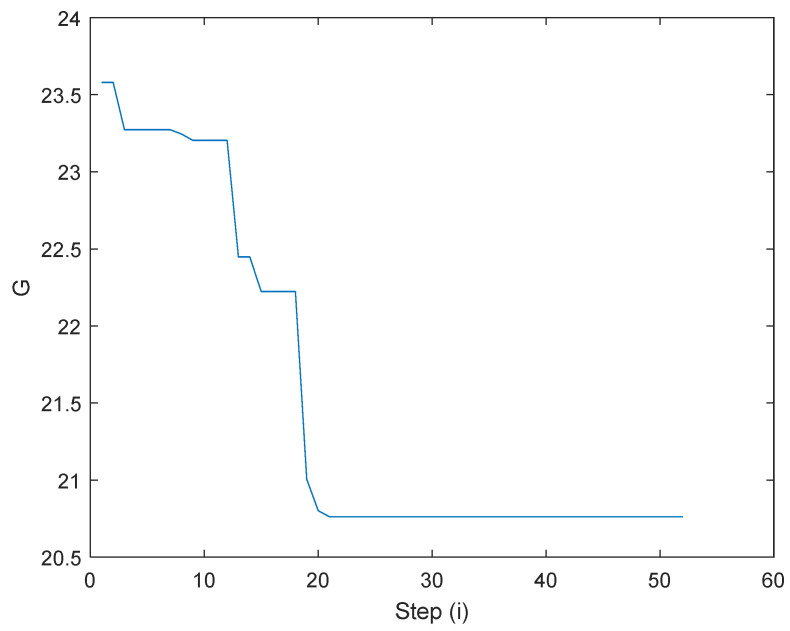
Algorithm converge.

All simulations are executed by using the combination of RECURDYN V8R4 and MATLAB R2019b. The RECURDYN works as a client to execute the dynamic environment; while MATLAB produces the electrical current to the landing gear system as a controller. Both software could share the data from solving the dynamic equation. After *N* = 52, the algorithm converges with value *G* to obtain the minimum at 20.7, as can be seen in Figure 14. The values of the optimal weight matrix and bias vector are given in Table 3.

## 6. Results and Discussion

In order to verify the strength of the proposed controller, a skyhook control, which is the most popular control to reduce vibration [14,24,25], is applied in this research as a competition. In this study, each landing gear is applied the skyhook control with the control law:

(25) $$F_{MR}=\left\{\begin{array}{c} C_{sky}\dot{s}\;if\;\dot{s}\dot{z}>0\\ 0\;if\;\dot{s}\dot{z}\leq 0 \end{array}\right.$$

**Figure 15:**
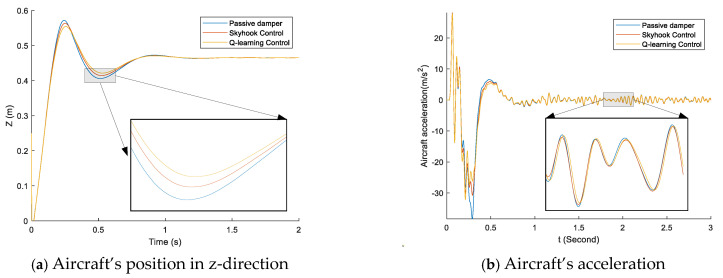

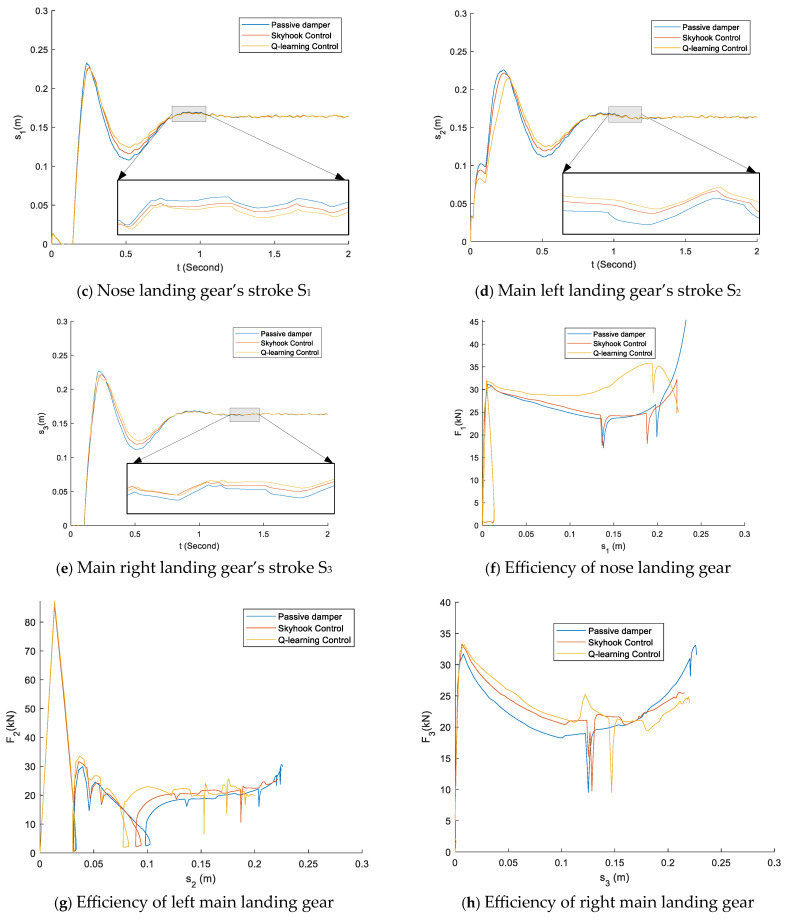
The comparison of the passive damper, skyhook control, and q-learning control in the case of one-point touchdown and sink speed of 3 m/s.

**Figure 16:**
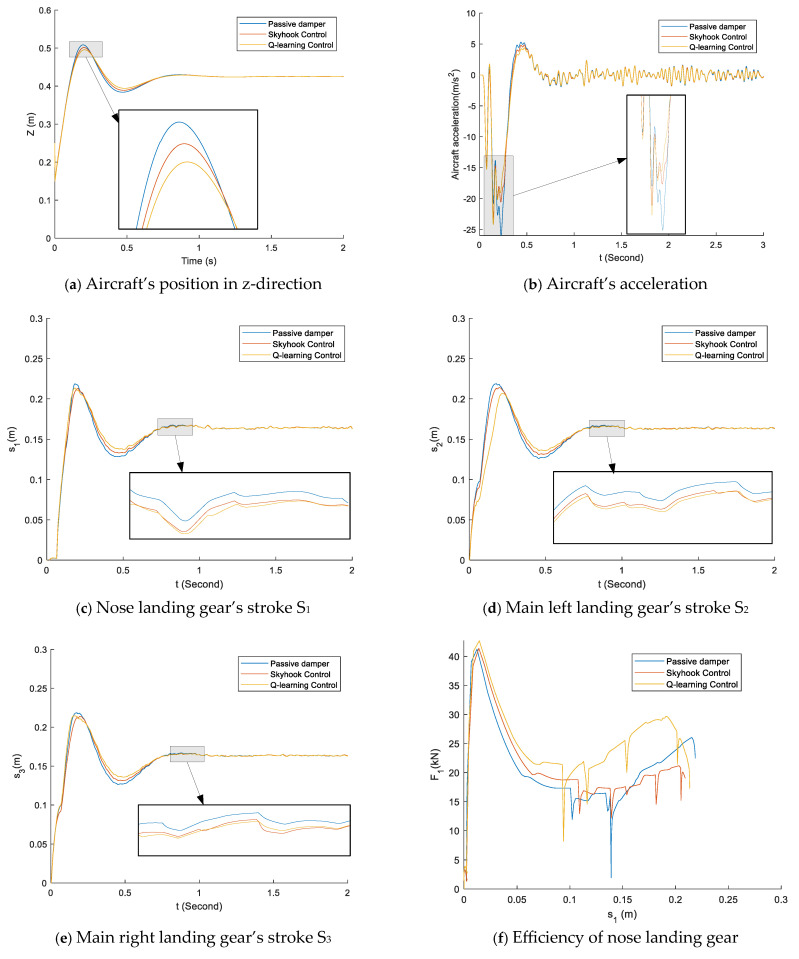

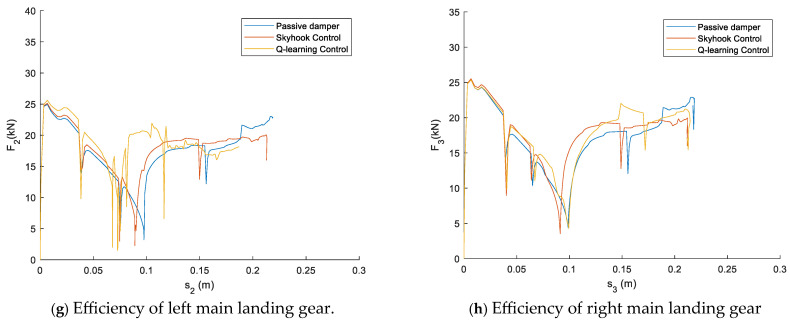
Comparison of the passive damper, skyhook control, and q-learning control in the case of two-point touchdown and sink speed of 3 m/s.

**Figure 17:**
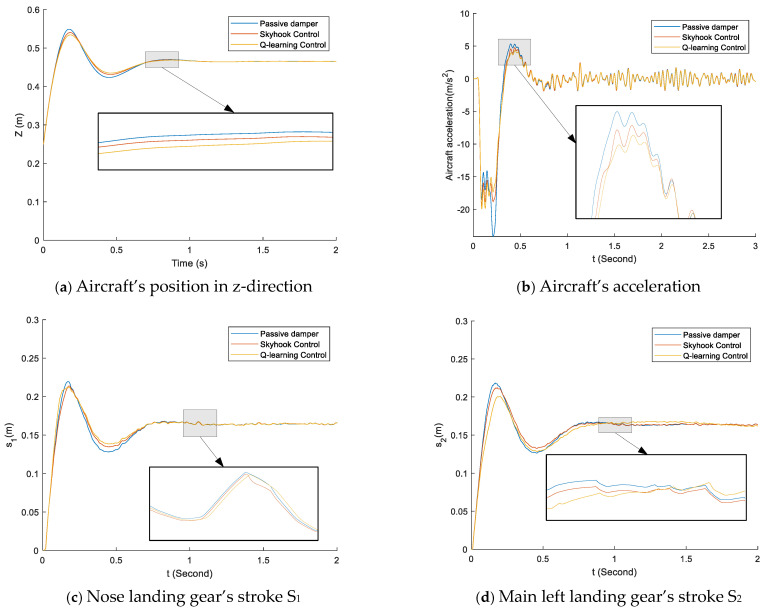

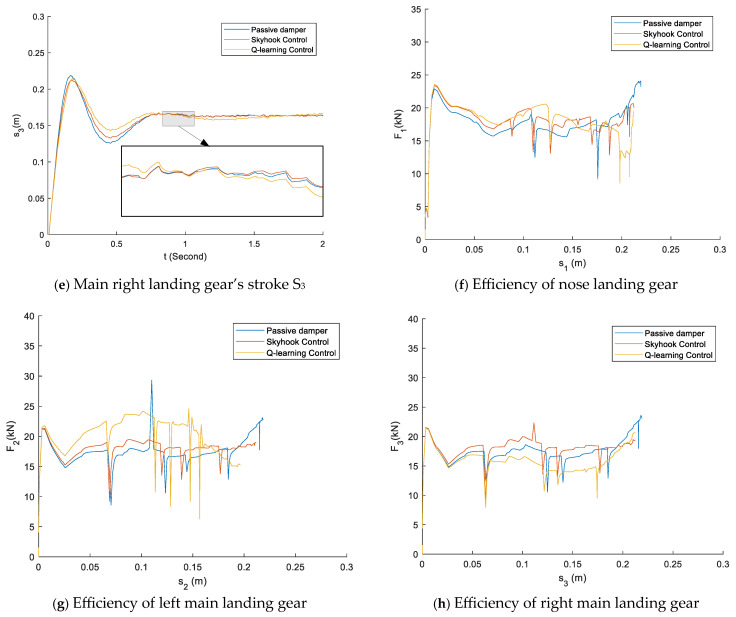
Comparison of the passive damper, skyhook control, and q-learning control in the case of a three-point touchdown and sink speed of 3 m/s.

The landing gear’s performance of one-point touchdown, two-point touchdown, and three-point touchdown are shown in Figure 15, Figure 16 and Figure 17, respectively. There are major vibrations in the landing gear’s stroke; however, a little vibration is shown in the aircraft’s position in the z-direction, so the landing gear did a good job of preventing the vibration from attaching to the aircraft’s body, as can be seen in Figure 15a, Figure 16a and Figure 17a. Figure 15 details the aircraft system response in the case of the one-point landing at 3 m/s sink speed. During touchdown, the left main landing gear makes contact with the ground first; around 0.2 s later, the right main landing touches the ground, and the nose landing gear makes contact with the ground the latest, after 0.3 s. The system provides better performance when it could reduce the small overshooting and go to the final state faster. The skyhook control and the proposed control decrease the peak value of acceleration and stroke more than the conventional passive damper, as can be seen in Figure 15a–e. The proposed control was fastest to get to the stable level. Moreover, the proposed control attempts to maintain the damping force during the first stroke, so it produced better shock absorber efficiency than the skyhook control.

The aircraft system response for a two-point landing at the sink speed of 3 m/s is shown in Figure 16. The nose landing strikes the ground around 0.1 s after the main landing gears make contact with the ground during touchdown. As shown in Figure 16a–e, the suggested control reduces the acceleration and stroke peak values more than the traditional passive damper and skyhook control. Furthermore, the suggested control outperformed the skyhook control in terms of shock absorber efficiency because it aimed to preserve the damping force during the initial stroke.

Figure 17 shows the response of the aircraft system during a three-point landing at a landing speed of 3 m/s. At the same time, the three landing wheels touch the ground. The recommended controller reduces the acceleration and peak stroke values more than the conventional passive damper and the skyhook controller, as shown in Figure 17a–e. Furthermore, because it reduces the maximum damping force in the initial stroke, the recommended controller performs better in terms of damping efficiency than the skyhook controller.

Moreover, the simulation results in various sink speeds and different landing phases, which are one-point touchdown (1-P), two-point touchdown (2-P), and three-point touchdown (3-P), are shown in Table 4. The skyhook control reduced a lot of vibration during the taxing phase. The maximum reduction in RMS is nearly 20% compared to that of the passive damper in the case of two-point landing and 3 m/s sink speed. In this case, the proposed control was reduced by only 15% compared to the passive damper. However, the proposed control improved the shock absorber efficiency in all simulation cases, the maximum shock absorber efficiency of the proposed control is nearly 86% during three-point landing and 1 m/s sink speed. While the passive damper only provides 81% and the skyhook control performs 60%. Thus, the proposed control provided better performance compared to the passive damper and skyhook control.

## 7. Conclusions

The paper adopts a landing gear system equipped with an MR damper that can improve the shock absorber efficiency in the touchdown phase and reduce aircraft acceleration during the taxing mode. Reinforcement learning based on Q-learning is invested to improve the shock absorber efficiency and to reduce the RMS in different landing scenarios involving rough ground. In order to verify the effectiveness of the controller, the co-simulations based on RECURDYN-MATLAB of the proposed system and the classical skyhook controller are executed. The proposed controller provides better performance compared to the skyhook controller and passive damper. In the future, the aircraft landing gear system will eventually be built to handle complex scenarios using other reinforcement learning techniques, such as deep learning. Also, the dynamic system will be developed, involving more complex noise and disturbances, such as wind and sensor noise.

## Figures and Tables

**Table 1:** Previous research in MR landing gear.

Author	Article	Scope of Study of MR Landing Gear	Control Strategies	Year
Jo et al.	[14]	Single landing gear, touchdown phase	On–off control, skyhook control, hybrid control	2021
Kang and Choi	[11]	Single landing gear, touchdown phase	On–off control, skyhook control, hybrid control	2024
Han et al.	[24]	Single landing gear, touchdown phase	On–off control, skyhook control,	2019
Kang et al.	[12,13,18,25]	Single landing gear, 6-DOF aircraft landing gear system, touchdown phase	On–off control, skyhook control, hybrid control, landing efficiency control,	2019–2023
Hao et al.	[16]	Single landing gear, touchdown phase		2023
QV Luong et al.	[21,26,27]	Single landing gear, touchdown phase	Supervise learning, genetic algorithm neural network, robust adaptive SMC	2020–2021
Yoon et al.	[17]	Single landing gear, touchdown phase	Energy conservation control,	2020
Le et al.	[28]	Single landing gear, touchdown phase	Model predicted control	2024
Lee et al.	[29]	Single landing gear, taxing phase	Skyhook control	2019
Present work		6-DOF aircraft landing gear system involving both touchdown phase and taxing phase	Q-learning neural network	

**Table 2:** Aircraft landing parameters.

Symbol	Value	Unit
*A_p_*	2.6 × 10^−3^	m^2^
*C*	7.0	kNs/m
*g*	9.81	m/s^2^
*M*	2250	kg
*m*	18	kg
*n*	1.3	
*k_T_*	412	kN/m
*V*_0_	6.29 × 10^−4^	m^3^
*u*	0~1	A
*v*(0)	1–3	m/s
*l_r_*	1.5	m
*l_l_*	1.5	m
*l_a_*	1.73	m
*l_b_*	0.87	m
*I_xx_*	79.1	kgm^2^
*I_yy_*	2030.6	kgm^2^
*I_zz_*	2317.2	kgm^2^
*φ*	0–7	°
*θ*	0–7	°

**Table 3:** Optimal weight matrix and bias vector.

W	0.07	0.01	0.18	−0.05	0.01	−0.10	−0.12	0.40	−0.09	0.06	0.00	0.25	0.06	−0.21	−0.04
	−0.11	0.25	0.20	−0.35	0.14	0.30	0.05	−0.31	−0.38	−0.45	0.24	−0.08	0.03	−0.04	−0.10
	0.09	−0.31	−0.15	−0.39	0.08	0.18	−0.09	−0.26	−0.10	−0.13	−0.08	0.34	−0.27	−0.35	−0.33
	0.01	0.45	0.15	−0.37	−0.20	−0.11	0.28	−0.17	−0.10	0.33	0.19	−0.52	0.27	0.10	0.18
	0.18	−0.19	−0.14	0.21	−0.14	0.35	−0.02	0.38	−0.20	0.10	−0.40	−0.45	−0.34	0.19	−0.02
	−0.10	−0.22	0.33	−0.09	−0.14	−0.14	0.47	0.04	0.38	0.09	0.15	0.47	0.44	−0.42	−0.01
	−0.11	−0.06	0.01	0.21	−0.42	0.34	0.37	0.17	0.21	0.18	0.48	−0.10	−0.27	0.38	−0.28
b	−0.51	0.02	−0.09												

**Table 4:** Landing performance using the passive damper (no control), skyhook controller, and Q-learning controller.

Case	Sink Speed (m/s)	Passive Damper				Skyhook Controller				Q-Learning Controller			
		*η*_1_	*η*_2_	*η*_3_	RMS	*η*_1_	*η*_2_	*η*_3_	RMS	*η*_1_	*η*_2_	*η*_3_	RMS
1-P	1	0.80	0.61	0.73	4.23	0.65	0.5	0.54	3.59	0.8	0.81	0.75	3.85
	2	0.70	0.43	0.74	5.3	0.69	0.46	0.6	4.39	0.82	0.61	0.76	4.78
	3	0.62	0.40	0.68	7.0	0.76	0.47	0.66	5.83	0.78	0.60	0.75	6.3
2-P	1	0.73	0.84	0.83	2.11	0.45	0.60	0.60	2.16	0.73	0.78	0.85	2.10
	2	0.63	0.79	0.77	3.11	0.49	0.60	0.60	2.97	0.66	0.73	0.78	2.87
	3	0.69	0.75	0.75	4.93	0.55	0.69	0.68	4.19	0.65	0.77	0.80	4.4
3-P	1	0.80	0.83	0.81	1.66	0.47	0.53	0.53	1.59	0.8	0.83	0.86	1.59
	2	0.76	0.86	0.87	2.84	0.5	0.6	0.6	2.88	0.78	0.77	0.85	2.68
	3	0.73	0.75	0.73	4.83	0.64	0.75	0.75	4.36	0.86	0.82	0.75	4.4

## Data Availability

The original contributions presented in this study are included in the article. Further inquiries can be directed to the corresponding author.

## Abbreviations

The following abbreviations are used in this manuscript:

$\dot{s}$	Stroke velocity
$\ddot{z}$	Aircraft body’s acceleration
*A_p_*	Cross-area of the piston
*b*, *b_f_*	Bias vector
*C*	Viscous damping coefficient
*Csky*	Skyhook factor
*D*	Draft force
*F_a_*	Pneumatic force
*F_d_*, *FMR_d_*, *FML_d_*, *FN_d_*	Damping force
*F*_max_	Maximum damping force
*F_MR_*	MR force
*F_T_*_1_; *F_T_*_2_; *F_T_*_3_	Reaction tired force
*Fv*	Viscous force
*g*	Gravitational acceleration
*G*, *G_passive_*	Cost function
*I*	Electrical current
*I_xx_*	Roll mass moment of inertia
*I_yy_*	Pitch mass moment of inertia
*I_zz_*	Yaw mass moment of inertia
*k_E_*	MR force factor
*k_T_*	Tire force constant
*L*	Lift force
*l_a_*	Distance between the nose landing gear interface and the aircraft’s center of gravity
*l_b_*	Distance between the main landing gear interface and the aircraft’s center of gravity
*l_l_*	Distance between the left main landing gear interface and the aircraft’s center of gravity
*l_r_*	Distance between the right main landing gear interface and the aircraft’s center of gravity
*M*	Aircraft mass
*m*	Landing gear mass
*n*	Polytropic process index
*N*, *N_repeat_*	Number of the simulation loops
*n_step_*	Number of sample signals
*RMS*	Root mean square
*s*	Landing gear stroke
*s*_max_	Maximum stroke
*T*	Engine thrust
*u*	Control input (electrical current)
*v*(0)	Initial sink speed
*V*_0_	Initial air chamber volume
*W*, *W_f_*	Weight matrix
*Wg*	Aircraft gravity force
*z*_1_, *z*_1_, *z*_3_	Tire deformation
*η*, *η*_1_, *η*_2_, *η*_3_	Shock absorber efficiency
*θ*	Pitch angle
*φ*	Roll angle
